# Congenital Zika Syndrome and Extra-Central Nervous System Detection of Zika Virus in a Pre-term Newborn in Mexico

**DOI:** 10.1093/cid/ciy616

**Published:** 2018-09-04

**Authors:** Maria Yolotzin Valdespino-Vázquez, Edgar E Sevilla-Reyes, Rosalia Lira, Martha Yocupicio-Monroy, Elvira Piten-Isidro, Celia Boukadida, Rogelio Hernández-Pando, Juan David Soriano-Jimenez, Alma Herrera-Salazar, Ricardo Figueroa-Damián, Gustavo Reyes-Terán, Rodrigo Zamora-Escudero, Jorge Arturo Cardona-Pérez, Angélica Maldonado-Rodríguez, Elsa Romelia Moreno-Verduzco, Jesús Miguel Torres-Flores

**Affiliations:** 1 Departamento de Anatomía Patológica, Instituto Nacional de Perinatología, Ciudad de Mexico, México; 2 Departamento de Investigación en Enfermedades Infecciosas, Instituto Nacional de Enfermedades Respiratorias, Ciudad de Mexico, México; 3 Unidad de Investigación Médica en Enfermedades Infecciosas y Parasitarias, Unidad Médica de Alta Especialidad Hospital de Pediatría, Centro Médico Nacional Siglo XXI, Instituto Mexicano del Seguro Social, Ciudad de Mexico, México; 4 Universidad Autónoma de la Ciudad de México, Posgrado en Ciencias Genómicas, Ciudad de Mexico, México; 5 Sección de Patología Experimental, Departamento de Patología, Instituto Nacional de Ciencias Médicas y Nutrición Salvador Zubirán, Ciudad de Mexico, México; 6 Departamento de Infectología e Inmunología, Ciudad de Mexico, México; 7 Dirección Médica, Ciudad de Mexico, México; 8 Dirección General, Instituto Nacional de Perinatología, Ciudad de Mexico, México; 9 Laboratorio de Virología, Escuela Nacional de Ciencias Biológicas, Instituto Politécnico Nacional, Ciudad de Mexico, México; 10 Subdirección de Servicios Auxiliares de Diagnóstico, Instituto Nacional de Perinatología, Ciudad de Mexico, México

**Keywords:** Zika virus, congenital Zika syndrome, co-infection, dissemination

## Abstract

**Background:**

During pregnancy, the Zika virus (ZIKV) replicates in the placenta and central nervous system (CNS) of infected fetuses; nevertheless, the ability of ZIKV to replicate in other fetal tissues has not been extensively characterized.

**Methods:**

We researched whether dissemination of congenitally-acquired ZIKV outside the CNS exists by searching for the accumulation of the viral envelope protein, ZIKV ribonucleic acid (RNA), and infectious viral particles in different organs of a deceased newborn with Congenital Zika Syndrome. A real-time qualitative polymerase chain reaction (qPCR) was used to detect ZIKV RNA in the brain, thymus, lungs, kidneys, adrenal glands, spleen, liver, and small intestine. The same tissues were analyzed by indirect immunofluorescence and immunoperoxidase assays using the monoclonal antibody 4G2 to detect ZIKV envelope antigens. Isolation of infectious ZIKV in a cell culture was carried out using brain and kidney samples.

**Results:**

A postmortem, virological analysis of multiple organs, such as the kidneys (epithelial cells in the renal tubules), lungs (bronchial epithelia), thymus (epithelial cells inside the Hassall’s corpuscles), and brain (neurons, ependymal cells, and macrophages) revealed the presence of ZIKV RNA and envelope antigens. Other tissues of the deceased newborn tested positive by qPCR for Epstein-Barr virus and human herpesvirus 6, including the brain cortex (Epstein-Barr) and the thymus, kidneys, and adrenal glands (human herpesvirus 6). The kidneys were identified as a significant niche for viral replication, given that infectious particles were successfully isolated from renal tissues.

**Conclusions:**

Our findings demonstrate the ability of congenitally-acquired ZIKV to produce disseminated infections and the viral tropism towards epithelial cells.

## Teaser

This is a detailed study that demonstrates the ability of congenitally-acquired Zika virus to disseminate outside a fetal central nervous system. Moreover, viral replication in the kidneys of the deceased newborn is also demonstrated by viral isolation and electron microscopy.

Zika virus (ZIKV) is a mosquito-borne *Flavivirus* transmitted to humans mostly through the bite of female *Aedes* mosquitoes. Since the first Zika fever cases reported in the Americas in early 2015, more than 200000 cases of ZIKV infection were reported in more than 50 countries in Latin America and the Caribbean by October 2016 [1]. The clinical presentation of ZIKV infection is mild or asymptomatic in approximately 80% of the infected individuals; nevertheless, during pregnancy, infections with ZIKV have been associated with Congenital Zika Syndrome (CZS), identified by a series of congenital neurological anomalies that include severe microcephaly and cerebral cortex thinning, as well as symptoms like seizures, irritability, and other central nervous system (CNS) disorders associated with brainstem dysfunction, such as feeding difficulties, hearing loss, and impaired vision [2–5].

ZIKV replicates and persists in the placenta, as well as in the fetal brain [6, 7]; however, the ability of this virus to replicate and persist in other human tissues remains unclear. Several studies have suggested that ZIKV has a wide cellular tropism and that other organs outside the CNS can become infected [8–10]. Amongst these organs, the kidney is particularly remarkable, because prolonged ZIKV shedding in the urine of infected patients has been observed, suggesting that this virus might have the ability to persist in renal tissues, as has been demonstrated with other flaviviruses [11–13].

Recently, ZIKV replication was observed in the epithelial cells of the proximal renal tubules of immunodeficient mice, as well as in primary human renal proximal tubular epithelial cells [14]. Moreover, other types of primary human renal cells, such as the renal glomerular endothelial cells and the mesangial cells, are also permissive to ZIKV replication [15]. ZIKV ribonucleic acid (RNA) has been detected in the renal tissues of human fetuses with CZS, supporting the theory that viral replication in renal tissues might be a common trait of flaviviral infections [16, 17]. Nevertheless, the sole presence of viral RNA is not enough to demonstrate that ZIKV can replicate in the human kidney.

In this work, we searched for ZIKV RNA and antigens in the kidney and other organs of a deceased newborn with CZS. Viral isolation from renal tissues provided evidence that the kidneys are active sites of ZIKV replication in congenitally-infected fetuses. Arbovirus and herpesvirus co-infections were also determined.

## METHODS

### Case History

In August 2016, a 22-year-old woman from Southern Veracruz, Mexico, developed a mild febrile illness at 14 weeks of gestation, accompanied by headaches, a skin rash, and general pruritus that lasted for 3 days. In October 2016, at 24 weeks of gestation, a prenatal evaluation with fetal ultrasonography revealed intrauterine growth restriction, and she was referred to the Instituto Nacional de Perinatología in Mexico City. At 28 weeks of gestation, further fetal ultrasonographic analyses confirmed the diagnosis and showed microcephaly, enlarged lateral ventricles, and cortical calcifications, as well as oligohydramnios. Maternal serum and amniotic fluid samples collected at 28 weeks of gestation tested negative for Zika, Dengue, and Chikungunya viruses RNA by real-time quantitative polymerase chain reaction (RT-qPCR). In late November 2016, at 30 weeks of gestation, the patient was admitted with severe preeclamptic symptoms, which led to a Cesarean section. The newborn died 4 hours after delivery due to Respiratory Distress Syndrome. The necropsy was performed with the written informed consent of the mother.

### Necropsy

The necropsy was performed 2 hours after the newborn’s death. Fresh tissue samples from different organs were collected and stored at -80°C for virological studies. Samples from the cerebral cortex, thymus, lungs, kidneys, adrenal glands, spleen, liver, and small intestine were fixed in 10% formalin and embedded in paraffin for further histopathological examination and for in situ detection of viral antigens.

### Virological Analysis

RT-qPCR assays were used to detect viral RNA from the cerebral cortex, thymus, lungs, kidneys, adrenal glands, spleen, liver, and small intestine. RT-qPCR amplification and melting curve profiles for arbovirus detection (Zika, Dengue, Chikungunya, West Nile virus; Sevilla-Reyes et al., in preparation) were used as indicators of related infections. All necropsy samples were also tested by qPCR for deoxyribonucleic acid (DNA) of the 8 human herpesviruses of medical importance (Herpes Simplex virus type 1 [HSV-1], Herpes Simplex virus type 2 [HSV-2], Varicella Zoster virus [VZV], Epstein-Barr virus [EBV], human cytomegalovirus [CMV], human herpesvirus [HHV]6, HHV7, and HHV8). Complete methodologies are provided in the Methods section of the Supplementary Appendix.

### Next-generation Sequencing

A brain sample was fragmented and passed through an insulin needle. The homogenate was clarified by centrifugation and filtered with a 0.45 µm syringe filter. The filtrate was digested with Turbo DNase (Ambion) and RNase I (Invitrogen) to remove non-protected human nucleic acids. Remaining nucleic acids were extracted using the QIAamp viral RNA minikit (Qiagen). Purified RNA was reverse-transcribed with Superscript III reverse transcriptase (Invitrogen) and second-strand DNA synthesis was performed with Klenow fragment polymerase (New England Biolabs) using barcoded primers consisting of a 20-nucleotide–specific sequence upstream of a random nonamer, as previously described [18]. The resulting DNA products were PCR amplified, and libraries were constructed with the Nextera XT DNA library preparation kit (Illumina) and sequenced on a NextSeq Illumina platform (2 × 150 bp run).

### Immunofluorescence and Immunoperoxidase Assays

The immunofluorescence and immunoperoxidase assays for the detection of ZIKV envelope antigen were carried out using the mouse anti-flavivirus envelope protein monoclonal antibody 4G2 [19], obtained from mouse immune ascitic fluid and an Alexa-555 goat anti-mouse immunoglobulin G (IgG) as a secondary antibody (Jackson ImmunoResearch) or rabbit anti-mouse antibodies labelled with horseradish peroxidase (Vectastain ABC Sytem, Burlingame, CA). Complete methodologies are provided in the Methods section of the Supplementary Appendix.

### In Situ Apoptotic Cell Detection

For in situ apoptotic cell detection, we used the DeadEnd peroxidase colorimetric apoptosis detection system kit (Promega), using brain tissue sections as described in the Methods section of the Supplementary Appendix.

### Ultrastructural Studies

For ultrastructural evaluation, small tissue fragments from the kidney cortex were analyzed by immunoelectronmicroscopy with the monoclonal antibody 4G2 and a rabbit anti-mouse IgG conjugated to 5 nm gold particles. Complete methodologies are provided in the Methods section of the Supplementary Appendix.

### Virus Isolation and Identification

A brain sample was fragmented, suspended, and homogenized in serum-free Dulbecco′s Modified Eagle Medium. Clarified supernatants were passed through 0.22 μm filters and used to infect Vero and C6/36 cell monolayers, which were then incubated at 37^o^C or 28°C, respectively, in a 5% CO_2_ atmosphere for 7–10 days. The supernatants of the infected Vero and C6/36 cells were recovered, and then 4 additional passages of the possible isolates were carried out in Vero cells. The identity of the ZIKV isolates was verified by RT-PCR and Sanger sequencing, as described in the Methods section of the Supplementary Appendix.

## RESULTS

**Figure 1.**
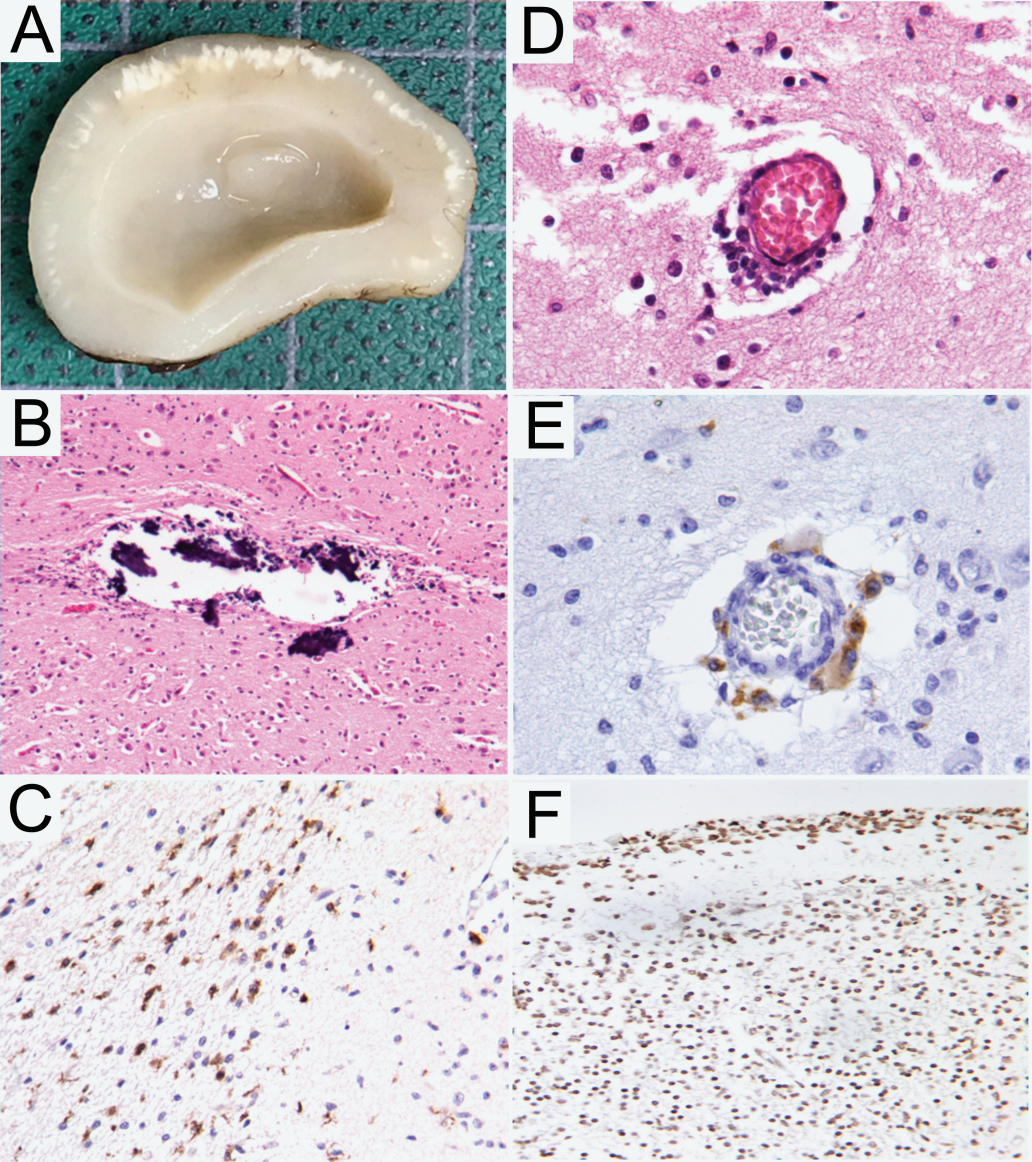
Central nervous system findings associated with Congenital Zika Syndrome. Cross sections of the brain display symmetric lateral ventricle enlargement and diffuse cortical and subcortical calcifications, as well as cortical mantle thinning, which was more accentuated towards the (*A*) temporal lobe. (*B*) The cerebral cortex is shown with extensive calcifications. (*C*) Numerous activated microglial cells were found near to the calcifications. (*D*) Occasional capillaries showed some inflammatory cells in the perivascular area. (*E*) Some of these inflammatory perivascular cells were macrophages that showed F4/80 positive immunostaining. (*F*) The TUNEL technique showed numerous apoptotic cells in cortical and subcortical areas.

Clinically-diagnosed CZS was confirmed by the pathological examination of the deceased newborn, both macroscopically and microscopically. External body examination of the 30 week–gestation female newborn revealed microcephaly with a head circumference of 23.5 cm (below the third percentile), micrognathia and retrognathia, low-set ears, and a depressed nasal bridge, as well as arthrogryposis. A macroscopic evaluation of the encephalic region revealed microcephaly, with a brain weight of 46.8 grams (Z-score: -5.35). The malformed brain showed an smooth outer cortical surface, with a total lack of gyri (lissencephaly). The cerebral lobes and the brain stem were hypoplastic. A cross-sectional examination of the brain revealed bilateral ventricular enlargement and a slim ribbon of cortical and subcortical white radial calcifications that were more prominent towards the occipital lobes (Figure 1).

A microscopic examination of the brain sections revealed extensive granular calcifications in the cortical and subcortical white matter, which were surrounded by activated microglia, as shown by ionized calcium binding adapter molecule 1 immunostaining (Figure 1). After extensive revision of the histological sections, we found occasional capillaries with minimal or mild inflammatory cells in the perivascular spaces, many of them positive to F4-80, a marker of peripheral macrophages that indicates monocyte emigration from capillary lumens to perivascular spaces (Figure 1). In the same sections, we performed the TUNEL technique to detect apoptotic bodies and found many apoptotic cells, particularly in the cerebral cortex (Figure 1).

ZIKV RNA was detected in the brain (cerebral cortex), thymus, lungs, kidneys, adrenal glands, and spleen, all of which were negative for other arboviruses (Table 1). The cerebral cortex and liver also tested positive for EBV infection, while the thymus, kidneys, adrenal glands, and liver samples tested positive for HHV6 (Table 1). Both the liver and proximal small intestine samples were negative for ZIKV RNA.

**Table 1.** Results From the Qualitative Polymerase Chain Reaction Assays for Detection of Arboviruses and Herpesviruses on Multiple Tissue Samples

	Cerebral Cortex	Lung	Thymus	Kidney	Adrenal Gland	Spleen	Liver	Small Intestine
**ZIKV**	POSITIVE (13.0)^a^	POSITIVE (18.3)a	POSITIVE (20.4)a	POSITIVE (21.2)a	POSITIVE (23.5)a	POSITIVE (27.6)a	-	-
**DENV** **1–4**	-	-	-	-	-	-	-	-
**CHIKV**	-	-	-	-	-	-	-	-
**WNV**	-	-	-	-	-	-	-	-
**HSV-1**	-	-	-	-	-	-	-	-
**HSV-2**	-	-	-	-	-	-	-	-
**VZV**	-	-	-	-	-	-	-	-
**EBV**	POSITIVE^b^	-	-	-	-	-	POSITIVE	-
**CMV**	-	-	-	-	-	-	-	-
**HHV6**	-	-	POSITIVE	POSITIVE	POSITIVE	-	POSITIVE	-
**HHV7**	-	-	-	-	-	-	-	-
**HHV8**	-	-	-	-	-	-	-	-

Abbreviations: CHIKV, Chikungunya virus; CMV, human cytomegalovirus; DENV, Dengue virus; EBV, Epstein-Barr virus; HHV, human herpesvirus; HSV, Herpes Simplex virus; VZV, Varicella Zoster virus; WNV, West Nile virus; ZIKV, Zika virus.

^a^Cq values

^b^Detected in next-generation sequencing.

Additionally, the complete genome of ZIKV (ZIKV/H.sapiens/Mexico/INPER38b/2016) was recovered from the brain tissue (accession number MG494697). A phylogenetic analysis of the complete genome sequence revealed that it clustered together with sequences collected from Mexico and Central America since late 2015, suggesting that they were still circulating in the region (Supplementary Figure 1) in mid-2016.

**Figure 2.**
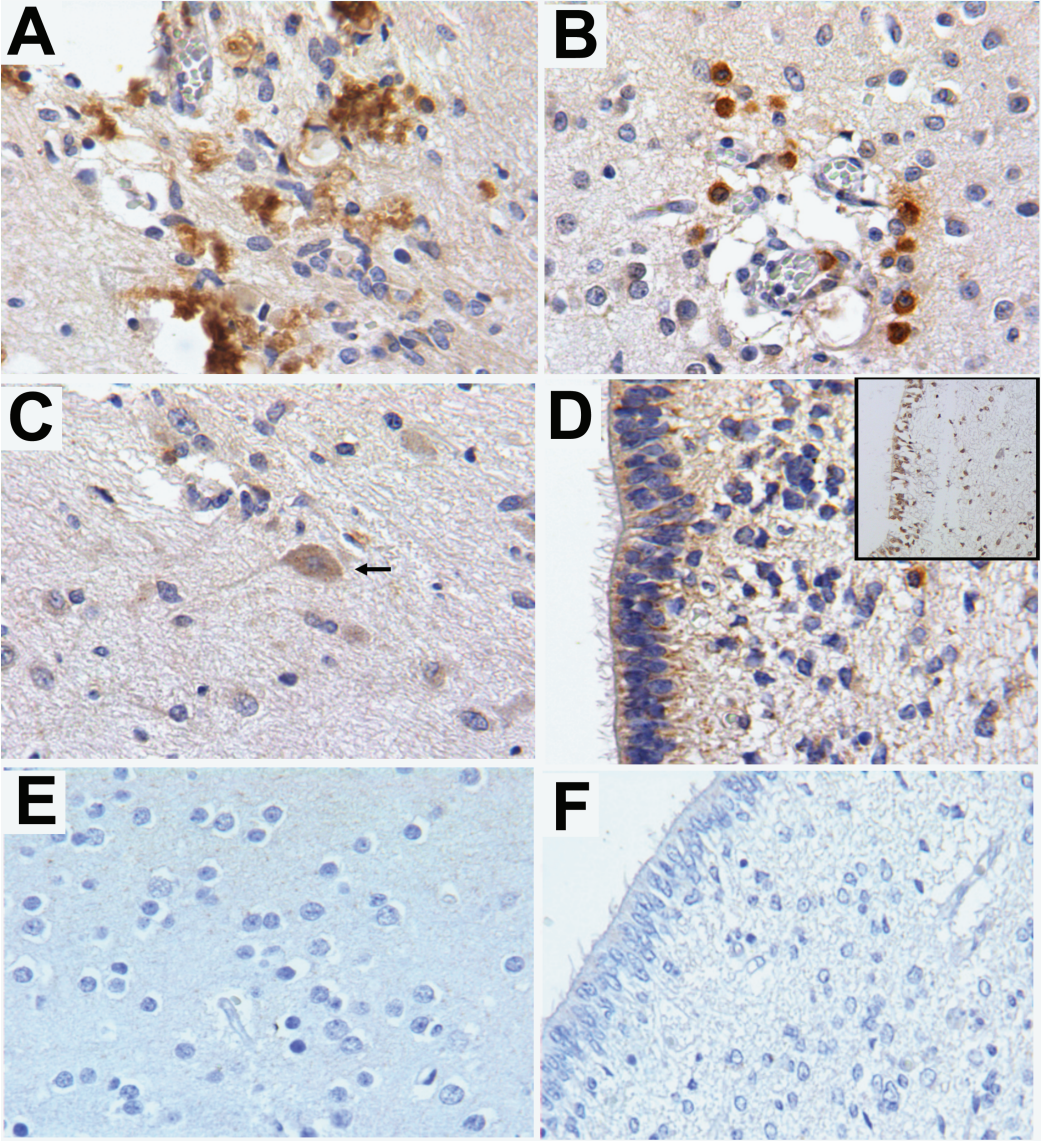
Detection of envelope antigens of the Zika virus in brain sections. Representative micrographs of Zika virus detection by immunoperoxidase with the antibody 4G2 in tissue paraffin–embedded fetal tissues showed (*A*) strong viral envelope (E) protein immunostaining around the calcified areas. (*B*) Macrophages around a brain capillary showed strong E protein immunostaining. (*C*) Occasional neurons showed mild E protein immunostaining (arrow). (*D*) Ependymal cells showed slight E protein immunostaining; additionally, the TUNEL technique showed numerous apoptotic bodies in the ependymal epithelium (inset). (*E* and *F*) Negative controls of the assays were performed in tissues from a non-infected newborn.

To analyze whether the presence of viral RNA in the tissues of the deceased newborn were accompanied by viral antigens, cross sections from organs that tested positive for ZIKV RNA (Table 1) were analyzed by immunoperoxidase and immunofluorescence staining for the viral envelope (E) protein using the monoclonal antibody 4G2. Viral antigens were not detectable in the spleen or adrenal glands, even though they had previously tested positive for viral RNA. In contrast, in the brain cortex there was strong immunostaining to the E protein around the calcified areas and in macrophages localized in perivascular infiltrates (Figure 2). Additionally, mild E protein immunostaining was observed in some neurons and in ependymal cells from the lateral ventricles, which also tested positive for apoptosis with the TUNEL technique (Figure 2). We also provide examples of negative controls in Figure 2.

**Figure 3.**
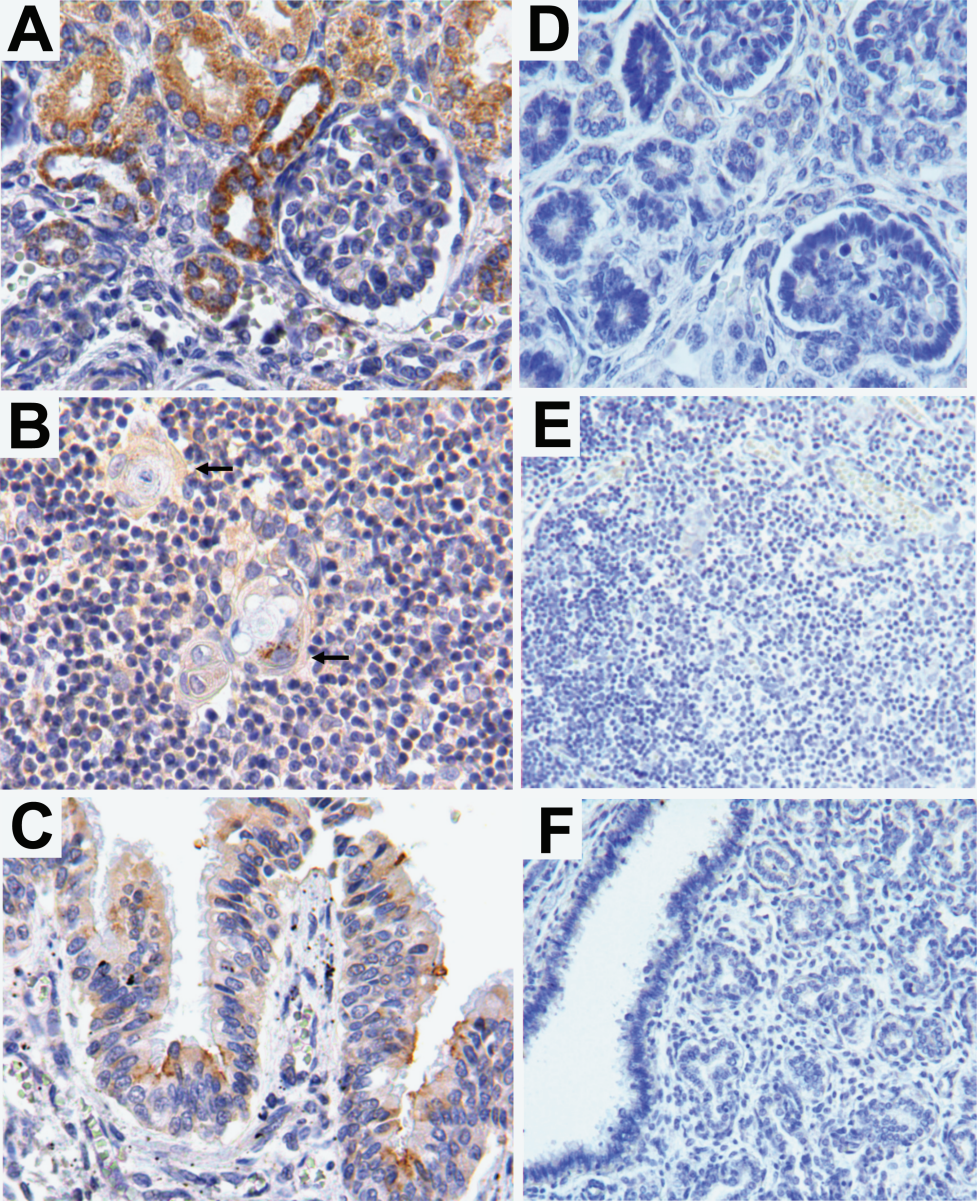
Detection of Flavivirus envelope protein in different fetal tissues by immunoperoxidase assay. Immunoperoxidase assay with the antibody 4G2 in tissue paraffin–embedded fetal tissues showed that (*A*) the epithelium from the cortical proximal and distal kidney tubules had strong envelope protein immunoreactivity. (*B*) Epithelial cells from the Hassall corpuscles in the thymus medulla showed slight E protein immunostaining (arrows), as did (*C*) the bronchial epithelial lumen. Negative controls of the assays were performed in tissues from a non-infected newborn in the (*D*) kidney, (*E*) thymus, and (*F*) lung.

**Figure 4.**
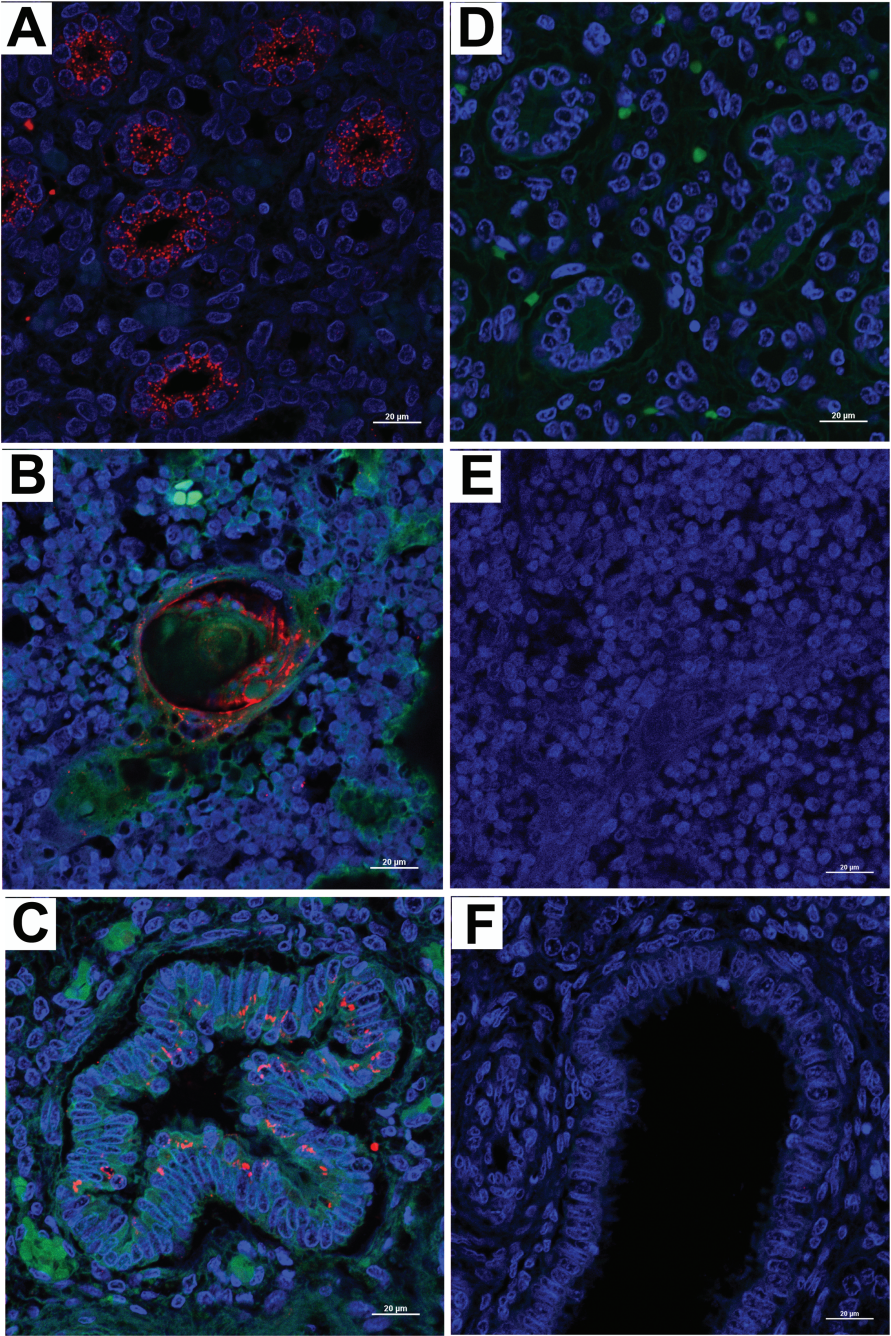
Detection of the Flavivirus envelope protein in different fetal tissues by immunofluorescence assay. Immunofluorescence assay with the antibody 4G2 in tissue paraffin–embedded fetal tissues showed (*A*) immunostaining in the epithelial cells from cortical convoluted tubules in the kidney and HassalĹs corpuscles in (*B*) the thymus and (*C*) the bronchial epithelial cells of the lung. Controls of the assays were performed in tissues from a non-infected newborn in the (*D*) kidney, (*E*) thymus, and (*F*) lung.

In the kidneys, strong immunostaining was detected in both the medullar and cortical tubules (Figures 3 and 4). Immunofluorescence detection of the E protein was preferentially located in the apical cytoplasm of tubular epithelial cells (Supplementary Video 1).

In the thymus, the E protein was only detected in the epithelial cells located inside the Hassall’s corpuscles, while no specific signal was observed in the cortex or in other histological elements of the medulla of the thymus (Figures 3 and 4). Finally, in the lungs, strong immunostaining was observed in the cytoplasm of the bronchial epithelial cells (Figures 3 and 4), preferentially displaying an apical localization.

**Figure 5.**
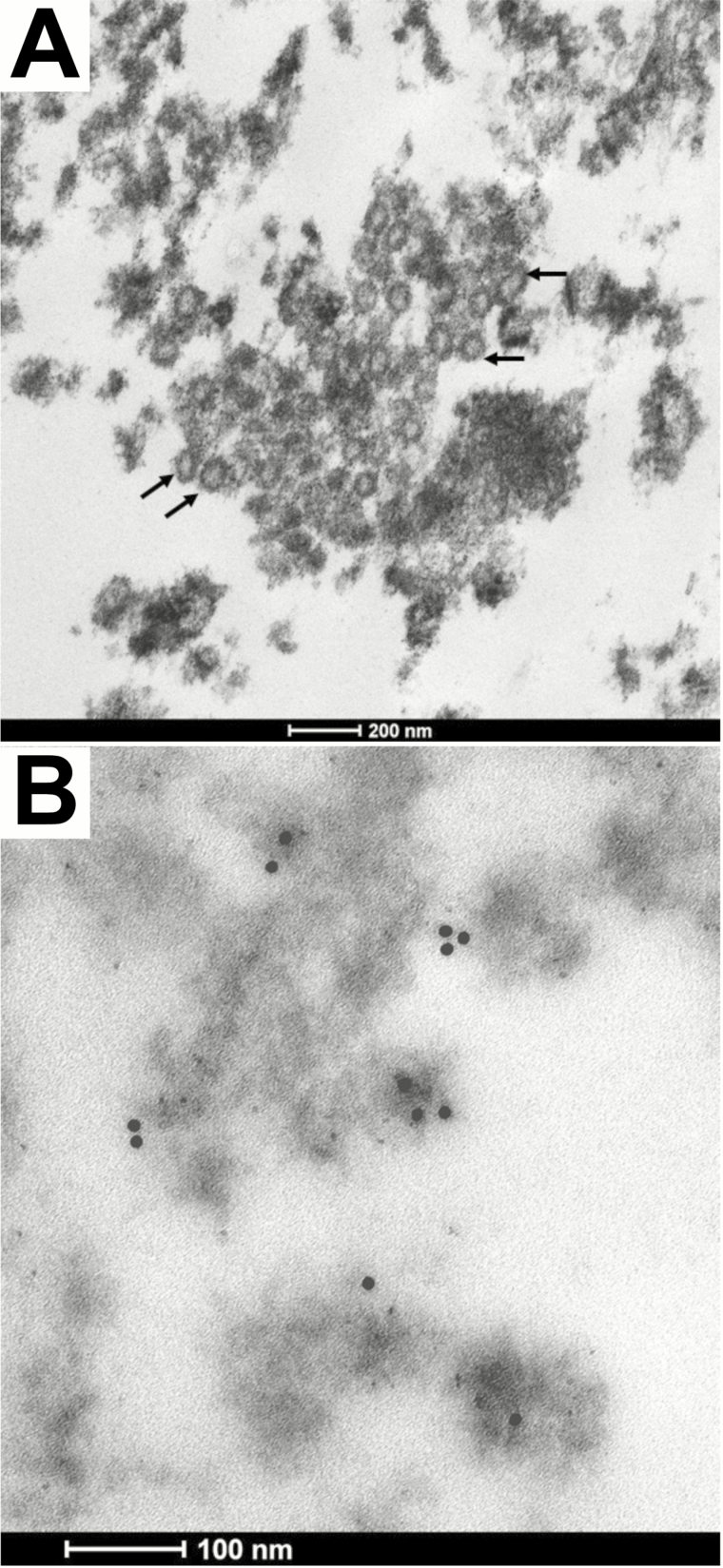
Virological analysis of kidney tissues. Representative electron microscopy micrographs of infected epithelial cells from the kidney. Panel *A* shows numerous virions in the cytoplasm of an epithelial cell from the proximal convoluted tubule (arrows); Panel *B* shows immunogold particles (black dots) on spherical electron dense structures, corresponding to virions in the epithelial cell from a proximal convoluted cell.

The high levels of ZIKV antigens and RNA detected in the brain, kidney, and lungs made us hypothesize that the virus was actively replicating in these organs. Virion-like particles were observed in sections of renal tissues by electron microscopy (Figure 5), suggesting the presence of ZIKV in the kidney of the deceased newborn. To confirm the presence of infectious viral particles in the kidney, as well as in the brain and lungs, virus isolation procedures were performed using fresh-frozen tissues. After 4 passages in Vero cells, the infected monolayers displayed a mild cytopathic effect, characterized by cell rounding and detachment. The RT-qPCR results suggested that the isolation of ZIKV was positive from the brain and kidney, but not from the pulmonary tissues (Table 2). To confirm the identity of the isolates, partial sequences of the E gene were recovered from the supernatants of the infected cultures showing a single synonymous substitution, in comparison to the viral sequences obtained by next generation sequencing directly from the brain cortex (Supplementary Figure 2).

**Table 2.** Results From the Real-Time Qualitative Polymerase Chain Reaction Assays for Identification of the Isolates of Zika Virus Obtained From Brain and Kidney Samples After 4 Passages in Vero Cellsa

	Positive control^b^	Brain (Vero)	Kidney (Vero)	Lung (Vero)
**ZIKV**	17.53	33.21	35.88	Negative

^a^Table displays Cq values

^b^For the positive control, RNA was extracted from Vero cells infected with the Zika virus reference strain PRVABC59.

## DISCUSSION

Several studies have demonstrated the link between intrauterine ZIKV infections and the development of a distinctive pattern of birth defects, grouped as CZS, that includes microcephaly and multiple fetal malformations such as arthogryposis and intrauterine growth restriction [3, 16], characteristic of the Fetal Akinesia Deformation Sequence. Moreover, common histopathological findings in the CNS tissues of autopsied fetuses and neonates with CZS include microcalcifications in the cortical and subcortical white matter, reactive gliosis, glial degeneration [3, 20] and, in some cases, perivascular lymphocytic cuffing with macrophage and mononuclear cell infiltrates [20, 21]. Nevertheless, the dissemination of ZIKV to other human fetal tissues has only been partially characterized.

The disseminative potential of ZIKV has already been demonstrated in human patients under corticosteroid therapy or with underlying medical conditions such as autoimmune diseases. In such cases, ZIKV RNA has been detected postmortem in the blood, brain, spleen, liver, kidneys, lungs, and heart [22, 23]. Additionally, infectious ZIKV was isolated from a pool of macerated organs (heart, lungs, and kidneys), suggesting that either of them might represent an important niche for ZIKV replication in immunosuppressed adult humans [24].

In several CZS cases, postmortem detection of ZIKV RNA in different tissues has been assessed by RT-PCR or in situ hybridization methods; still, some of them have failed to detect viral RNA outside the fetal CNS and the placenta [3, 25, 26]. In an analysis that was among the first to detail a CZS case, the presence of viral RNA was reported in the spleen, lungs, liver, and muscle, suggesting the possible dissemination of ZIKV to multiple fetal tissues [27]. Later, during the ZIKV outbreak in the Americas, the presence of ZIKV RNA was reported in the spleens, kidneys, and livers of 2 fatal cases of CZS in Colombia [28]. Further analyses of the tissues of 7 deceased neonates in Brazil demonstrated that ZIKV RNA was present in the brains, lungs, hearts, livers, spleens, and kidneys in 3 of the cases studied [29], supporting the theory that disseminated infections might be occurring in some cases of CZS. Moreover, in situ hybridization analyses of fetal human tissues have also demonstrated that the liver, kidneys, and spleen might be active sites of ZIKV replication in fetuses [16]. Consistent with previous findings, this report provides evidence of the presence of ZIKV RNA in multiple fetal tissues, suggesting that disseminated infections might be affecting different organs in each case; however, due to technical issues, retrospective study comparisons could be difficult.

The dissemination of ZIKV into multiple fetal tissues has also been evaluated by the immunohistochemical detection of viral antigens: mainly by the detection of the viral E protein [30] with the pan-flaviviral antibody 4G2. To date, the presence of the E protein of ZIKV has been reported in brain tissues, as well as in placental tissues [20], chorionic membranes, and umbilical cords [17]. The presence of the E protein has been reported in neurons, in areas of microcalcification in the brain parenchyma, in degenerating glial cells, and in microglia and endothelial cells [21]. In this report, we were also able to detect ZIKV antigens in cells from the ventricular ependymal epithelium. The implications of ZIKV infection of ependymal cells in humans remain unclear; nevertheless, it seems that the viral infection could induce apoptosis, considering that we found many TUNEL-positive ependymal cells [31, 32].

Interestingly, the presence of ZIKV antigens in systemic organs during congenital infections has only been reported in animal models [10, 33] despite several attempts to detect ZIKV E proteins in human fetal tissues using 4G2 [3, 20]. In this report, we could detect ZIKV antigens in multiple organs using the monoclonal antibody 4G2. In the brain, we detected the E protein in cells from the ventricular ependymal epithelium and in macrophages located in perivascular spaces. The strong immunoreactivity displayed by peripheral macrophages towards F4/80 and the ZIKV E protein supports the hypothesis that hematogenous dissemination of the virus might play a role in the development of intrauterine ZIKV infections with multi-organ involvement.

The presence of the viral-like particles in the renal tissues and the isolation of infectious ZIKV from renal tissues demonstrate that the kidneys are an active site of ZIKV replication in the fetus. Moreover, the renal tubular epithelium appears to be at risk for ZIKV infection.

ZIKV replication in the kidneys might explain the continuous viral shedding observed in the urine of some congenitally-infected newborns [34]. Seemingly, viral excretion in urine could be a common trait of flaviviral infections [35], although viral isolation from the renal tissues of patients with flaviviral infections has not previously reported [36, 37]. Yet, several studies must be performed to rule out the possibility that ZIKV replication in fetal kidneys might entail a higher risk of renal damage.

We also detected ZIKV antigens in pulmonary tissues and in the Hassall’s corpuscles in the thymus, which is suggestive of ZIKV replication in these organs. Pulmonary hypoplasia, interstitial lymphocytic pneumonitis, and expansion of the alveolar septa have all been reported as part of the clinical manifestation of CZS in fetuses [29], and have been hypothesized to be a consequence of viral replication in pulmonary tissues. Even though were unable to isolate infectious ZIKV from the lungs of the infected fetus, we cannot rule out the fact that the lungs might support viral replication. Further, the clinical implications of ZIKV E protein in the thymus remain unclear and, even though the presence of viral antigens in the epithelial cells of the thymus is not conclusive enough to ensure that they can support viral replication, this finding increases the importance of studying the role of the thymus during intrauterine ZIKV infections, especially because of the important role that it plays during the establishment of self-tolerance in the fetus.

The presence of EBV and HHV6 in several tissues of the deceased newborn raises even more questions about the factors that might have an influence over congenital ZIKV infections. Even though both viruses have been previously associated with congenital infections [38, 39], further studies are needed to understand whether they have an influence in the extra-CNS dissemination of ZIKV, as well as over the severity of the disease.

Infections with HSV-2 have been shown to enhance ZIKV infection in placental tissues, demonstrating the possible role of members of the *Herpesviridae* family in the severe cases of CZS [40]. Many populations have high prevalences of pathogens like HHV6 [41, 42]; thus, the coinfection with ZIKV or with other arboviruses is likely. Still, the influence of latent infections with HHV over the course and severity of congenital ZIKV infections remains to be explored.

It is important to highlight that, as in previous reports [9, 17, 21, 29], no dramatic pathological changes were found in most of the tissues that were analyzed, confirming that ZIKV causes the most devastating damage to the fetal brain. However, these data may forecast other, not-so-obvious pathologies like renal damage, autoimmune diseases, or other postnatal complications that may arise in the future for congenitally-infected newborns.

In conclusion, we demonstrated the ability of ZIKV to replicate in the human kidney and disseminate to other organs in congenitally-infected fetuses. Moreover, we demonstrated that other congenital viral infections might co-exist with ZIKV, increasing the need to study them to analyze their role in the pathogenesis of congenital ZIKV infection.

## Supplementary Data

Supplementary materials are available at *Clinical Infectious Diseases* online. Consisting of data provided by the authors to benefit the reader, the posted materials are not copyedited and are the sole responsibility of the authors, so questions or comments should be addressed to the corresponding author.

Supplementary Appendix

Supplementary Video 1

## Notes

***Author contributions.*** M. Y. V.-V., E. E. S.-R., R. L., M. Y.-M., and R. H.-P. contributed equally as primary investigators in this article. The rest of the authors contributed substantially to the completion of this study and preparation of this manuscript.

***Acknowledgments.*** The authors thank the mother in this case for her willingness to provide detailed medical data. They also thank Vadim Pérez Koldenkova for his support with the confocal microscopy at the Laboratorio Nacional de Microscopia Avanzada/Centro Medico Nacional Siglo XXI, Instituto Mexicano del Seguro Social; Brenda Marquina for her collaboration in the TUNEL assays; Maria Loroño for the donation of the monoclonal antibody 4G2; and Othon Rojas Montes for his technical support. The authors thank the reviewers for their valuable comments on this manuscript. They thank Red INMUNOCANEI-CONACyT 269316 and Red Mexicana de Virología- CONACyT 269826.

***Financial support.*** This work was supported by grants from CONACyT (grant numbers 183448 and 233977).

***Potential conflicts of interest.*** E. E. S.-R. and R. H.-P. are partly supported by Red CONACyT INMUNOCANEI. All other authors report no potential conflicts. All authors have submitted the ICMJE Form for Disclosure of Potential Conflicts of Interest. Conflicts that the editors consider relevant to the content of the manuscript have been disclosed.
