# Supplementary material for: Congenital Zika Syndrome and Extra-Central Nervous System Detection of Zika Virus in a Pre-term Newborn in Mexico
Source: Clin Infect Dis. 2018 Sep 4;68(6):903–12. doi: 10.1093/cid/ciy616 (PMC6399440; doi:10.1093/cid/ciy616)
Supplement: Supplementary Appendix [file ciy616_suppl_supplementary_appendix.docx]

**Supplementary Appendix**

**Congenital Zika Syndrome and Extra-CNS detection of Zika virus in a pre-term newborn in Mexico**

Maria Yolotzin Valdespino-Vázquez, M.D., Edgar E. Sevilla-Reyes, Ph.D., Rosalia Lira, Ph.D., Jesús Torres-Flores, Ph.D., Martha Yocupicio-Monroy, Ph.D., Elvira Piten-Isidro M.Sc., Celia Boukadida, Ph.D., Rogelio Hernández-Pando, M.D., Juan David Soriano-Jimenez, M.D., Alma Herrera-Salazar, Ph.D., Ricardo Figueroa-Damián, M.D., Gustavo Reyes-Terán, M.D., Rodrigo Zamora-Escudero, M.D., Jorge Arturo Cardona-Pérez, M.D., Angélica Maldonado-Rodríguez, M.Sc., Elsa Romelia Moreno-Verduzco, M.D.*

**Contents**

1. Supplementary Video 1 2

2. Supplementary Figure 1 3

3. Supplementary Figure 2 4

4. Supplementary Methods 5

1. **Supplementary Video 1**

<https://www.dropbox.com/s/dg88ow6n0gtzc39/170220_Rinhon_Prim%2BSec_4G2-AF555_DAPI_autofl_60x_2epsi_5_3D%20-%20Movie.avi?dl=0>

**Supplementary Video 1. Time-lapse laser-scanning confocal fluorescence microscopy (3D movie) of flavivirus E protein detection in renal tubules**. 3-D confocal fluourescence microscopy reconstruction of ZIKV detection in renal tubules. Immunofluorescence assay with panflavivirus monoclonal antibody 4G2, secondary antibody anti-IgG-Alexa fluor 555. Magnification 60X. 22 optical sections of 0.5 μm were used to construct de 3-D video. Images were acquired on a Nikon Ti Eclipse inverted confocal microscope equipped with an A1 imaging system

1. **Supplementary Figure 1**

**Supplementary Figure 1. Complete Zika virus genome phylogenetic reconstruction.** INPER38b (*) was located close to other Mexican and Central American viruses. Five hundred trees were built, using all complete ZIKV genomes available in NCBI by the end of February 2017, in RaxML software (A. Stamatakis: "RAxML Version 8: A tool for Phylogenetic Analysis and Post-Analysis of Large Phylogenies".  Bioinformatics, 2014) using a Maximum-Likelihood method with GTR+gamma as a substitution model. The tree with the highest likelihood is shown with bootstrap confidence values near the nodes.

1. **Supplementary Figure 2**

INPER38BRAIN 1 ATACTGCTGATTGCCCCGGCATACAGCATCAGGTGCATAGGAGTCAGCAATAGGGACTTT

Brain_culture 1 ............................................................

Kidney_culture 1 ............................................................

consensus 1 ************************************************************

INPER38BRAIN 61 GTGGAAGGTATGTCAGGTGGGACTTGGGTTGATGTTGTCTTGGAACATGGAGGTTGTGTC

Brain_culture 61 ............................................................

Kidney_culture 61 ............................................................

consensus 61 ************************************************************

INPER38BRAIN 121 ACCGTAATGGCACAGGACAAACCGACTGTCGACATAGAGCTGGTTACAACAACAGTCAGC

Brain_culture 121 ............................................C...............

Kidney_culture 121 ............................................C...............

consensus 121 ********************************************.***************

INPER38BRAIN 181 AACATGGCGGAGGTAAGATCCTACTGCTATGAGGCATCAATATCAGACATGGCTTCGGAC

Brain_culture 181 ............................................................

Kidney_culture 181 ............................................................

consensus 181 ************************************************************

INPER38BRAIN 241 AGCCGCTGCCCAACACAAGGTGAAGCCTACCTTGACAAGCAATCAGACACTCAATATGTC

Brain_culture 241 ............................................................

Kidney_culture 241 ............................................................

consensus 241 ************************************************************

INPER38BRAIN 301 TGCAAAAGAACGTTAGTGGACAGAGGCTGGGGAAATGGATGTGGACTTTTTGGCAAAGGG

Brain_culture 301 ............................................................

Kidney_culture 301 ............................................................

consensus 301 ************************************************************

INPER38BRAIN 361 AGCCTGGTGACATGCGCTAAGTTTGCATGCTCCAAGAAAATGACCGGGAAGAGCATCCAG

Brain_culture 361 ............................................................

Kidney_culture 361 ............................................................

consensus 361 ************************************************************

INPER38BRAIN 421 CCAGAGAATCTGGAGTACCGGATAATGCTGTCAGTTCATGGCTCCCAGCACAGTGGGATG

Brain_culture 421 ............................................................

Kidney_culture 421 ............................................................

consensus 421 ************************************************************

INPER38BRAIN 481 ATCGTTAATGACACAGGACATGAAACTGATGAGAATAGAGCGAAGGTTGAGATAACGCCC

Brain_culture 481 .........................-----------------------------------

Kidney_culture 481 ............................................................

consensus 481 *************************...................................

INPER38BRAIN 541 AATTCACCAAGAGCCGAAGCCACCCTGGGGGGTTTTGGAAGCCTAGGACTTGATTGTGAA

Brain_culture ------------------------------------------------------------

Kidney_culture 541 ............................................................

consensus 541 ............................................................

INPER38BRAIN 601 CCGAGGACAGGCCTTGACTTTTCAGATTTGTATTACTTGACTATGAATAACAAGCACTGG

Brain_culture ------------------------------------------------------------

Kidney_culture 601 ............................................................

consensus 601 ............................................................

INPER38BRAIN 661 TTGGTTCACAAGGAGTGGTTCCACGACATTCCATTACCTTGGCACGCTGGGGCAGACACC

Brain_culture ------------------------------------------------------------

Kidney_culture 661 ............................................................

consensus 661 ............................................................

INPER38BRAIN 721 GGAACTCCACACT

Brain_culture -------------

Kidney_culture 721 .............

consensus 721 .............

**Supplementary Figure 2:** Sequence alignment of the M/E coding region from cultured viruses found in brain and kidney (Sanger sequencing) compared to the virus sequences (NGS) found directly in the brain cortex. A single synonymous substitution was observed. Dots in the alignment indicate identity to the top sequence, asterisks indicate conserved residues in all sequences, while dashes mark unobtained sequence.

1. **Supplementary Methods**

**Virological analysis**

Viral RNA was extracted using the QIAmp Viral RNA Mini Kit (QIAGEN) after pre-treatment with proteinase K (QIAGEN). Briefly, the genetic material and a multiplexed cocktail of primers were added to a mastermix of the OneStep RT-PCR kit (QIAGEN) for arboviruses or of the PCR Multiplex kit (QIAGEN) for herpesviruses for 15 cycles of PCR. The BioMark (Fluidigm) platform and the 48.48 dynamic array IFC (Fluidigm) were used for parallel nano-qPCR amplification and melting curve detection using the SsoFast EvaGreen Supermix with Low ROX (Biorad).

**Immunofluorescence and immunoperoxidase assays**

The immunofluorescence and immunoperoxidase assays for the detection of ZIKV envelope antigen were carried out as follows. Serial sections (5 µm) from the brain, spleen, kidneys, thymus, adrenal glands, and lungs were deparaffinated, rehydrated, permeabilized with ice-cold methanol for 10 min at -20°C and then incubated with PBS containing 10% FBS and 0.2% Triton X-100 for 1 hour at room temperature. The tissue sections were washed twice with washing buffer (PBS containing 1% BSA) and incubated overnight at 4°C with the mouse anti-flavivirus envelope protein monoclonal antibody 4G2 (Henchal, 1982). Sections were then washed and incubated for 1 hour at room temperature with a 1:250 dilution of an Alexa-555 goat anti-mouse IgG antibody (Jackson ImmunoResearch). Sections were mounted using VECTASHIELD mounting media with DAPI (Vector Laboratories). Cells were analyzed by confocal microscopy (Nikon).

For immunohistochemistry, paraffin-embedded sections from the brain, kidneys, lungs and thymus were dewaxed and re-hydrated before blocking endogenous peroxidase with methanol-10% H_2_O_2_. Tissue sections were blocked with HBS with 2% Background Sniper (Biocare Medical) and then incubated overnight with the monoclonal antibody 4G2, anti-F4-80 (peripheral macrophage marker, eBioscience, San Diego, CA, USA), and Iba1 (activated microglia). Then, slides were incubated with rabbit anti-mouse antibodies labelled with horseradish peroxidase (AB/HRP) (Vectastain ABC Sytem, Burlingame, CA, USA). Finally, peroxidase reaction was revealed with diaminobenzidene/H_2_O_2_. Slides were washed and counterstained with Harris hematoxylin.

***In situ* apoptotic cell detection**

For in situ Brain tissue sections (5 μm thick) were mounted on silane-covered slides, refixed with 4% paraformaldehyde dissolved in PBS, and incubated for 10 min at room temperature with proteinase K (20 µg/mL) dissolved in Tris-HCl buffer. Tissue sections were covered with equilibration buffer (200 mM potassium cacodylate, 25 mM Tris-HCl, 0.2 mM DTT, 0.25 mg/ml BSA, 2.5 mM cobalt chloride; pH 6.6), followed by incubation with the cocktail solution (36 µl equilibrium buffer, 4 µl FITC-labeled UTP nucleotides, 1 µl recombinant terminal deoxynucleotidyl transferase) for 1 h at 37°C in a humid chamber. The sections were then washed with 2X SSC solution, and the endogenous peroxidase was quenched with 0.3% H_2_O_2_. Brain tissues were incubated with streptavidin coupled to peroxidase, revealed with diaminobenzidine, and counterstained with Harris hematoxylin. Negative controls consisted in incubating the sections with the equilibrium buffer solution plus nucleotide mix without terminal deoxynucleotidyl transferase enzyme.

**Ultrastructural studies**

Small tissue fragments from kidney cortex were obtained from the paraffin blocks, postfixed with 2% OsO_4_ buffer, dehydrated in graded ethyl alcohol solutions and embedded in Epon Resin (London Resin Company). Thin sections from 70 to 90 nm were contrasted with lead and uranium salts and examined with a FEI Tecnai G2 Spirit transmission electron microscope (Hillsboro, OR, USA). For immunoelectronmicroscopy, small fragments of the kidney cortex were dehydrated and embedded in LR-White hydro soluble resin. 70 to 90 nm sections were mounted on nickel grids and incubated overnight at room temperature with the monoclonal antibody 4G2. After extensive washing, rabbit anti-mouse IgG conjugated to 5-nm gold particles diluted 1/20 were incubated for 2 h at room temperature. The grids were contrasted with uranium salts and analyzed in an electron microscope.

**Virus isolation and identification**

To verify the identity of the ZIKV isolates, 10 mL of clarified supernatants from infected Vero cells (fourth passage) were concentrated by centrifugation at 4000 x g for 10 min using Amicon® Ultra 100K devices. Viral RNA was extracted from the concentrated viral lysates as previously described. The 5' coding region of the E protein was amplified by RT-PCR using two primers (Fw 5'GCTTGGCTTTTGGGAAGCTC3'; Rv 5' TTGGCATGTGCGTCCTTGAAC 3') to obtain a DNA product of 809 bp which was further purified and sequenced by the Sanger Method (Macrogen). Multiple sequence alignments were carried out using MAFFT version 7.
